# Current status and future directions in unresectable stage III non-small cell lung cancer

**Published:** 2020-10-29

**Authors:** Esperanza Arriola Arellano, Verónica Díaz Díaz, Joaquín José Cabrera Rodríguez

**Affiliations:** ^1^Department Medical Oncology, Universitary Hospital Puerta del Mar, Cádiz, Spain; ^2^Department of Radiation Oncology, Universitary Hospital Puerta del Mar, Cádiz, Spain; ^3^Department of Radiation Oncology, Universitary Hospital of Badajoz, Badajoz, Spain

**Keywords:** Non-small cell lung cancer, radiotherapy, immunotherapy, image-guided radiotherapy, intensity-modulated radiotherapy, radio-chemotherapy, advanced cancer

## Abstract

**Background:**

Patients with unresectable stage III non-small-cell lung cancer constitute a heterogeneous group in which the available treatments may range from radical therapies with radio-chemotherapy to supportive treatments depending on the extent of the disease and comorbidities present. For years the standard treatment based on the combination of chemotherapy and radiotherapy (RT) has remained unchanged and survival outcomes have been poor.

**Aim:**

Recent advances in molecular biology and RT technology have resulted in improved survival. This article reviews the treatments that constitute current standard treatment in unresectable advanced lung cancer and the situations and indications for the management of patients who are not candidates for radical therapy.

**Relevance for Patients:**

Although unresectable lung cancer does not have a good prognosis, new drugs and new technologies in radiation oncology can offer treatment options adapted to the patient’s clinical situation, ranging from therapies administered with radical intent to others aimed mainly at improving the patient’s quality of life, which, judiciously chosen, will provide optimal management of the patient.

## 1. Systemic Therapy

### 1.1. Introduction

Patients with unresectable stage III non-small-cell lung cancer (NSCLC) constitute a heterogeneous population. No single definition of “unresectable” at this stage of NSCLC is universally accepted. In general, resectability is determined on a case-by-case basis by an experienced thoracic surgeon in a multidisciplinary team environment. For more than a decade, no improvement had been achieved in outcomes for patients with unresectable locally advanced NSCLC (LA NSCLC). The standard treatment in that setting is definitive concurrent chemotherapy and radiation (CCRT), but while the intent of treatment is curative, most patients rapidly progress. Recently, in the PACIFIC trial, durvalumab consolidation therapy demonstrated a statistically significant improvement in progression-free survival (PFS) and in overall survival (OS). Here, we review the systemic treatment of unresectable LA NSCL [1].

### 1.2. Standard treatment

The current standard of care for these patients is CCRT. The recommendation to add chemotherapy to RT is based on studies showing an improved OS for that regimen compared with RT only, with a meta-analysis demonstrating an absolute benefit of 2.2% at 5 years (hazard ratio [HR]: 0.89; 95% confidence interval [CI]: 0.81-0.98; *P*=0.02) [2] Furthermore, chemotherapy administered concurrently is preferred to sequential treatment, given the significant OS benefit of 4.5% at 5 years (HR: 0.84; 95% CI: 0.74-0.95; *P*=0.004) [3].

The optimal concurrent chemotherapy regimen has not been determined. Commonly used combinations include cisplatin-etoposide and weekly low dose carboplatin-paclitaxel. Other chemotherapeutic schemes in the concomitant scenario have also emerged: Cisplatin/docetaxel, cisplatin/vinorelbine, and cisplatin/pemetrexed (non-squamous only). Studies show acceptable toxicity and relatively similar OS rates [1,4,5].

Grade 3 or 4 esophagitis occurs more frequently with CCRT than with sequential chemoradiation (SCRT). Patients should be selected on the basis not only of their anticipated response to therapy but also on how well they are expected to tolerate therapy. Accelerated RT regimens may be useful if CCRT might not be tolerated (this issue has been extensively discussed in another chapter). Finally, SCRT or radiotherapy (RT) alone is recommended for frail patients who cannot tolerate concurrent CCRT (Eastern Cooperative Oncology Group [ECOG] >0/1, or patients who have lost more than 5% of their usual body weight) [1,4].

### 1.3. Improving standard treatment

Most patients will relapse after CCRT. Median PFS is short at 8-12 months, and 5-year OS rates are still low at 15-25%. These values have remained relatively unchanged over time [1].

Given the high risk of metastasis and short PFS after CCRT, two strategies aimed at improving outcomes are induction chemotherapy before CCRT and consolidation therapy (defined as treatment administered after the end of a defined number of chemotherapy cycles with or without RT, in a patient whose tumor has been controlled). However, cancer and leukemia Group B 39801 trial evaluated 2 cycles of carboplatin area under the curve 6 and paclitaxel 200 mg/m^2^ administered every 21 days followed by CCRT and found that induction chemotherapy increased toxicity and provided no survival benefit over CCRT alone [6]. Moreover, a pooled analysis of 42 studies comparing consolidation chemotherapy after CCRT with best supportive care showed no difference in median OS: 19.0 months (95% CI, 17.3-21.0) and 17.9 months (95% CI, 16.1-19.9), respectively [7].

To date, no phase III trials studying consolidation chemotherapy with targeted treatment or vaccines have demonstrated a benefit in PFS or OS in patients with unresectable LA NSCLC. The SWOG SOO23 study examined gefitinib after CCRT and docetaxel consolidation, administered until progression or unacceptable toxicity, for up to 5 years. Despite a reasonable safety profile, OS was significantly lower in the gefitinib arm [8]. Another 2 studies examined the use of vaccine therapies for consolidation in this setting: The START trial comparing tecemotide (LBLP25) with placebo and the STOP trial comparing belagenpumatucel-L with placebo, both of which failed to show statistical improvements in OS [9,10]. More recent phase III studies (KCSG-LU05-04, PROCLAIM, and RTOG 0617) found that neither the addition of combination chemotherapy with cisplatin and docetaxel or pemetrexed nor the anti-EGFR antibody, cetuximab, to CCRT improved survival [11-13]. Similarly, increasing the dose of radiation to 74 Gy from the standard 60 Gy was not associated with a OS benefit (RTOG 0617); in fact, the standard treatment arm was shown to be superior, with a median OS of 20.3 months for patients receiving high-dose RT (HR: 1.38; 95% CI: 1.08-1.76; *P*=0.004) (Table 1) [13-15].

**Table 1:** Attempts to improve outcomes in unresectable stage III non-small-cell lung cancer.

Strategy	Study	Survival (months)
Increased radiotherapy dose	74 Gy versus 60 Gy; RTOG 0617 (2015)	20.3 versus 28.7 (*P*=0.004, detrimental)
Pre- or post-CCRT chemotherapy	Carbo/paclitaxel before CCRT; CALGB (2007)	12 versus 14 (*P*=0.3)
	Docetaxel following CCRT; HOG (2008)	21.2 versus 23.2 (*P*=0.883)
	Cisplatin/vinorelbine following CCRT; GILT (2016)	20.8 versus 18.5 (*P*=0.87)
	Cisplatin/pemetrexed following concomitant cisplatin/pemetrexed/RT; PROCLAIM (2016)	26.8 versus 25.0 (*P*=0.98)
	Cisplatin/docetaxel following CCRT; KCSG-LU05-04 (2015)	20.6 versus 21.8 (*P*=0.44)
Addition of targeted agents	Gefitinib consolidation; SWOG S0023 (2008)	23 versus 35 (*P*=0.013, detrimental)
	Cetuximab; RTOG 0617 (2015)	25 versus 24 (*P*= 0.29)
Vaccination post-CT/RT	Tecemotide; START (2014)	25.6 versus 22.3 (*P*=0.123)
	Belagenpumatucel-L; STOP (2015)	20.3 versus 17.8 (*P*=0.594)
Immuno checkpoint inhibitors	Durvalumab; PACIFIC (2017)	NR versus 28 (HR: 0.68; *P*=0.0025)
	Pembrolizumab; LUNG 14-179 (2018)	NR
	Phase II (single arm)	
	Atezolizumab; DETERRED (2018)	Part 1: 20.1
	Phase II (single arm)	Part 2: NR

### 1.4. Consolidation treatment with immunotherapy

The use of immune checkpoint inhibitors (ICIs) as consolidation therapy in a curative intent management plan for LA NSCLC represents a promising strategy to improve outcome after CCRT [16]. Durvalumab is a fully human monoclonal antibody directed against PD-L1 that blocks binding to its PD-1 and CD80 receptors, eliciting enhanced T cell activity against tumor cells. The PACIFIC trial [16], a phase 3 randomized trial, compared adjuvant treatment with durvalumab 10 mg/kg administered every 2 weeks (q2w) for 12 months (also known in this setting as consolidation immunotherapy) versus placebo in eligible patients with unresectable stage III NSCLC (PS 0-1) who had not progressed after treatment with 2 or more cycles of definitive concurrent platinum-based CCRT. Durvalumab was associated with a statistically significant and clinically meaningful improvement in survival at 2 years, with an absolute difference of 10.7% in the durvalumab arm (66%; 95% CI: 61.7-70.4%) compared to the placebo arm (55.6%; 95% CI: 48.9-61.8; *P*=0.0005). With a median follow-up of 25.2 months, the median OS had not been reached with durvalumab, while median OS with placebo was 28.7 months (HR: 0.68; *P*=0.0025) [17].

In an update of OS outcomes 3 years after the last patient was randomized (data cut-off January 31, 2019), the benefit of durvalumab in OS compared with placebo remained consistent (stratified HR 0.69, 95% CI, 0.55-0.86); median OS was not reached (95% CI, 38.4 months-NR) with durvalumab versus 29.1 months (95% CI, 22.1-35.1) with placebo. These updated results show that the clinical benefits of durvalumab in terms of OS are maintained in the longer term. Importantly, more than 50% of patients receiving durvalumab were alive at 36 months (specifically, 57.0% vs. 43.5% receiving placebo) [18].

Overall, treatment with durvalumab was well tolerated. The safety profile was consistent with previous reports from earlier studies. Grade 3 or 4 adverse events occurred in 30.5% (*n*=145) of patients in the durvalumab group and 26.1% (*n*=61) of patients in the placebo group. Rates of grade 3-5 pneumonitis were low in both arms, and no meaningful difference was observed (4.4% vs. 4.3%) [17].

Durvalumab thus fills a critical unmet need in the setting of unresectable LA NSCLC and provides a new option for patients treated with curative intent who do not progress on CCRT.

Other ICIs are currently under investigation for patients with unresectable LA NSCLC. Phase II studies of pembrolizumab and atezolizumab have demonstrated PFS and safety profiles similar to those seen with durvalumab, providing further support for the effectiveness of those anti–PD-1/PD-L1 antibodies in improving outcomes for those patients [19,20].

#### 1.4.1. Unanswered questions

Several questions remain unanswered, including the timing of immunotherapy (consolidation treatment versus concurrent with definitive CCRT), the selection of patients who will benefit most from immunotherapy, and, importantly, the identification of biomarkers (PD-L1 or others). The European Medical Agency currently authorizes consolidation with durvalumab only in patients with PD-L1 expression ≥1%, based on an unplanned post hoc analysis suggesting a lack of benefit with durvalumab in patients with PD-L1-negative tumors; however, this decision has been highly criticized by the scientific community. The safety and efficacy of durvalumab in populations that were not included in the PACIFIC trial are still unknown, for example, in patients with multiple comorbidities and poor performance status and patients who receive SCRT rather than CCRT [21]. In this tenor, the Spanish Lung Cancer Cooperative Group conducted the DURVAST study to explore the feasibility of durvalumab treatment in patients with advanced cancer and virologically controlled HIV-1 infection. This study demonstrated that durvalumab treatment was feasible and safe in HIV-1-infected patients with cancer receiving combination antiretroviral therapy [22].

### 1.5. Future directions

Further research aimed at optimizing the use of ICIs in LA NSCLC is currently investigating the timing and duration of treatment, and clinical trials are evaluating other immunotherapeutic agents such as pembrolizumab, nivolumab, ipilimumab, and atezolizumab.

The phase 3 PACIFIC 2 study assessed a fixed dose of durvalumab (1500 mg) every 4 weeks (q4w) (the schedule currently approved for the treatment of extensive-stage small-cell lung cancer in the United States [US]) [23], administered alongside CCRT [24]. Two studies, PACIFIC 5 (phase 3) and PACIFIC 6 (phase 2), are evaluating the same schedule of durvalumab administered as consolidation treatment following sequential CRT [25]. The PACIFIC 6 incorporates a cohort of patients with WHO/ECOG PS 2 (see NCT03693300 at clinicaltriasls.gov). The DUART study is evaluating durvalumab in patients with stage III disease and an ECOG PS of 0 to 2 who were treated with RT but are ineligible for chemotherapy (NCT04249362). The COAST trial is a phase 2, randomized, multidrug platform study designed to identify potential combinations of durvalumab with novel agents that improve response rates beyond those of monotherapy in the post-CCRT setting. Potential drugs to be evaluated must meet certain criteria. Oleclumab and monalizumab meet these criteria and will be used in the initial experimental arms of this study (NCT03822351). Finally, a novel USA study is investigating the effects of the combination of durvalumab and stereotactic body radiation therapy (SBRT) following CCRT in unresectable stage III NSCLC patients (NCT03589547).

Other clinical trials evaluating other immunotherapeutic agents are also currently in progress. One such study is the pembrolizumab phase 2 trial (NCT03379441), which is evaluating the use of CCRT followed by pembrolizumab maintenance (up to 24 months). The NICOLAS trial assesses the use of nivolumab given earlier in treatment by administering it concurrently with chemotherapy and radiation (NCT02434081). An interesting project in this setting is CheckMate73L, a phase 3, 3-arm trial, the primary purpose of which is to compare the effectiveness of nivolumab plus CCRT followed by nivolumab plus ipilimumab (arm A) versus CCRT followed by durvalumab (arm C) (NCT04026412). Two trials which evaluate the efficacy of induction chemoimmunotherapy are the KEYNOTE-799 trial, a phase 2 study that assesses first-line pembrolizumab plus chemotherapy given before CCRT plus pembrolizumab followed by pembrolizumab consolidation (NCT03631784) and, in a similar approach, a nivolumab phase 2 study (NCT04085250) which evaluates nivolumab consolidation therapy in patients who have not progressed following neoadjuvant chemotherapy plus nivolumab and definitive CCRT. Finally, a rather interesting phase 3 trial (KEYLYNK-012) is assessing the efficacy and safety of pembrolizumab in combination with CCRT followed by either pembrolizumab with olaparib placebo (Arm 1) or with olaparib (Arm 2) compared to CCRT followed by durvalumab (Arm 3) (NCT04380636).

Lastly, against the background of tumors that have activating EGFR mutations, the LAURA trial, is a phase 3 study which is evaluating the efficacy and safety of osimertinib following CCRT in patients with stage III EGFR mutation-positive NSCLC. CCRT may have been given either concurrently or sequentially. Patients whose disease has not progressed following CCRT have been randomized to receive osimertinib or placebo. The estimated primary completion date is July 2022 (NCT03521154).

### 1.6. Summary

The introduction of maintenance immunotherapy with the PD-L1 inhibitor durvalumab opened a new therapeutic window for stage III NSCLC patients who achieve at least stable disease after CCRT, as shown by the PACIFIC study. However, half of the patients still show disease progression at 18 months [16,17]. Those patients, therefore, represent a critical unmet need, warranting expedited approval of and access to new treatments that can improve outcomes.

## 2. Stage III Unresectable Non-small Cell Lung Cancer (NSCLC): The Blurred Line between Radical and Palliative Treatment

### 2.1. Introduction

The goal of treatment of the patient with unresectable stage III NSCLC who is not a candidate for CCRT is to maintain quality of life (QoL). The decision to treat should be supported by an accurate diagnosis and based on a disciplinary committee discussion. It appears that treating a highly selected group of unresectable stage III patients with platinum-based CCRT could have favorable outcomes for survival without compromising the QoL.

The key factors in decision making are:

- Disease-related: Tumor extension, existence of mutations, expression and percentage of PDL-1, histology, and nodal involvement.
- Treatment-related.
- Patient-related: Respiratory function, comorbidities, presence of symptoms, and general condition.

Management of patients with unresectable stage III disease who are not really fit for curative treatments is a very complex challenge because of the lack of high-quality scientific evidence. Standard radiation therapy may not be the best option for these individuals. In fact, altered fractionation, especially accelerated and hypofractionated schedules, may be more suitable [13,26]. Early data from retrospective or phase II studies with protons have suggested survival improvement in stage III patients with tolerable toxicity [27,28].

At present, age is not an independent criterion to drive treatment decisions. In a Japanese trial [29], patients over 70 years treated with CCRT had better OS compared to those treated with RT alone (OS; median, 22 vs. 17 months; HR 0.68, 95% CI 0.47-0.98). However, there is an increase in cardiac toxicity and a higher prevalence of comorbidities in older patients [30]. Nowadays, fit elderly patients are encouraged to receive CCRT [31].

The patient’s general condition is the main factor to determine the intention of treatment [32]. Treatment of individuals with ECOG >2 should be tailored according to the goals, either palliation of symptoms or stabilization of disease, and, in general, toxic schedules should be avoided.

The choice of palliative RT schedule will be based on the vital prognosis according to the patient’s performance status. Two systematic reviews [33,34] failed to demonstrate any differences between different palliative RT schemes in terms of efficacy or QoL; however, 2-year OS was higher for dose schedules BED_10_ greater than 35 Gy [34], so for patients with the better general condition, the most widely recommended schedule in use is 30 Gy in 10 fractions. Shorter and more hypofractionated schedules, such as 20 Gy/5f/d or 17 Gy/2f/w, are more useful for symptom control in patients with a higher ECOG score.

The American Society for Radiation Oncology published a clinical practice guideline on this topic [35]. Prognostic factors associated with worse outcome were tumor diameter >8 cm, forced expiratory volume <40%, weight loss > 10% in 6 months, and ECOG ≥2. It should be noted that this guideline was supported by studies that used technology now considered outdated and chemotherapy schedules that are no longer considered appropriate. Some studies did not report QoL data [35]. A recent study suggests that novel radiation therapy technology improves QoL without compromising local control [36]. Asymptomatic or minimally symptomatic patients who are not candidates for curative treatment do not benefit from immediate palliative treatment as opposed to deferred treatment [37].

Randomized studies of palliative RT in unresectable stage III NSCLC were summarized by Jumeau *et al*. [37] (Tables 2 and 3).

**Table 2:** Randomized studies of palliative radiotherapy in unresectable stage III NSCLC.

Studies	Patients (*n*)	Schedules	Results
Simpson 1985	316	40 Gy/20f versus	No difference
		30 Gy/10f versus	
		40 Gy/10 f split course	
Teo 1998	273	45 Gy/18 f versus	Palliation 71% versus 54% (*P*: 0.012)
		31.2 Gy/4 F/w	No difference in toxicity and survival
Abratt 1995	84	35 Gy/10 f versus	Symptom response 68% versus 76%
		45 Gy/15 f	1 y-OS 40% versus 37% esophagitis 23% versus 41%
MRC1991	369	30 Gy/10 f versus	No difference
		27 Gy/6f versus	
		17 Gy/ 2f/ 8 d	
MRC1992	235	17 Gy/2 F/8 d versus	Palliation 19% versus 64%
		10 Gy 1 f	Dysphagia 23% versus 56%
MRC 1996	509	36-39 Gy/12-13 f versus	Better palliation with 2 f
		17 G7/2 f/8 d	OS 2 y 12% versus 9% (0:0.003) More toxicity with 13 f
Rees 1997	216	17 Gy/2 f/8d versus	No difference
		22.5 Gy/5f/5 d	
Reinfuss 1999	240	50 Gy/25 f versus	MS: 12 versus 9 versus 6 m
		40 Gy/10 f split versus	OS 18% versus 6% versus 0% (*P*<0.05)
		20-25 Gy/4-5 f/d	
Nestlé 2000	152	32 Gy/16f BID versus	No difference
		60 Gy/30 f	
Bezjak 2002	230	20 Gy/ 5f versus	MS 6 versus 4.2 m (*P*: 0.0305)
		10 Gy/1 f	Better QLC-C30 with 5 F
Erridge 2005	148	30 Gy/10 f versus	MS: 28.3 versus 22.7 w
		10 Gy/f	Better chest pain control for 10 f
Kramer 2005	297	30 Gy/10 f versus	OS-1 y: 19.6% versus 10.9% (*P*: 0.03)
		16 Gy/2 f/8d	Longer palliation with 10 f
Sundstrom 2004	407	17 Gy/2f/8d versus	No difference
		42 Gy/15 f versus	
		50 Gy/25 f	
Senkus-Konefka 2005	100	20 Gy/5 f versus	MS: 5.3 m versus 8 m (*P*:0.016)
		16 Gy/2 f/8d	

d: Day; f: Fraction; MS: Median survival; OS: Overall survival; w: Week

**Table 3:** Studies with concomitant chemotherapy versus palliative radiotherapy in advanced stage III NSCLC [37].

Studies	Patients (*n*)	Schedules	Results
Ball 1997	200	RT (20 Gy/5 f) versus CCT (5FU)	6 m versus 6.8 m esophageal toxicity 3 versus 12%
Nawrocki 2010	99	RT (30 Gy/10 f) versus CCT(CPDD+VNB)	9 m versus 12.9 m (*P*:0.034) Toxicity G3 0 versus 2%
Strom 2013	191	CT (CARBO+VNB)	9.7 m versus 12.6m (*P*<0.01) 1.3 versus 30%
		versus CCT (42 Gy/15 f)	HRQOL worse for CT only

CARBO: Carboplatin; CCT: Concomitant chemo-radiotherapy; CPDD: Cisplatin; CT: Chemotherapy; f: Fraction; FU: Fluorouracil; m: Month; RT: Radiotherapy; VNB: Vinorelbine

### 2.2. Relapse after external RT in unresectable lung carcinoma

Local recurrence after RT remains a major challenge despite advances in systemic and RT treatments. Contemporary radiation techniques such as volumetric and image-guided RT (IGRT) and the possibility of increasing dose per fraction may help to overcome tumor radioresistance, thus opening the door to the possibility of reirradiation with better results.

Selection of patients is critical since most also present distant relapses or poor general condition, so only palliative RT will be indicated. Reirradiation can improve symptom control in case of hemoptysis or superior vena cava syndrome, but not in the case of dyspnea [38].

#### 2.2.1. Radical reirradiation by conventional RT

Reirradiation using conventional RT requires a balance of potential toxicity and benefits. The existing studies were based on phase I/II retrospective and prospective single-center studies, mixed histologies, and different doses of RT with and without added chemotherapy [39]. Extreme caution should be taken to avoid radiation therapy-related severe adverse effects such as pneumonitis, bronchial fistulas, and esophageal perforation [40]. Caution must be taken with overlapping radiation fields, especially in centrally located tumors, where there is a higher probability of long-term toxicity [41].

Wu *et al*. [42] found some factors linked to better outcomes: A disease-free interval (DFI) of more than 6 months, PS ≥70%, FEV1 >1 L. Other researchers also found that a longer time between the first course of the RT and reirradiation was correlated with higher survival [43].

2.2.1.1. Radical reirradiation by stereotactic ablative RT (SABR)

The available evidence on the efficacy and security of SABR in the reirradiation setting is derived from retrospective studies. The published results are promising [44], with local control rates of up to 86%, a progression-free interval of 30%, and a mean OS of 14-22 months.

Better prognosis was found when tumor volumes were <75 cm^3^, in second primary tumors, and in patients with PS > 80%. Toxicity was related to poor PS, mediastinal radiation therapy, and FEV1 <65% [44].

SABR reirradiation is preferentially recommended for peripheral lesions because of the concern of severe toxicity in central tumors. Death secondary to SABR has been described due to massive hemoptysis or aortoesophageal fistula, but may also be related to disease progression [44].

In conclusion, reirradiation is a valid tool for both salvage and palliative retreatment depending on the individual risk-benefit balance of the patients, some of whom benefit from higher doses of RT; high-precision conformal treatments should be performed in all cases. Maximum caution should be taken with central tumors due to the increased risk of toxicity, but dose constraints for organ at risk (OAR) in the reirradiation setting are not yet clearly defined. There are a number of prognostic factors that can help in the decision to perform treatment:

- Small tumor volumes.
- Good PS.
- Good respiratory function.
- DFI more than 12 months.

### 2.3. Radiomics, an emerging tool in predictive models

In the era of personalized medicine, specifically in the field of oncology in NSCLC, the current goal is more individualized treatment. To that end, the accuracy of diagnosis at the molecular and biological level must be improved.

Lung cancer is a disease that develops due to multiple genetic mutations which translate into inter- and intra-tumoral heterogeneity, in which the tumor microenvironment is fundamental to tumorigenesis. These aspects were not evident in conventional imaging studies, and radiomics could be the strategy that solves this problem.

Radiomics is an emerging non-invasive technology that uses image analysis to acquire quantitative information automatically or semi-automatically to obtain a large amount of data that can be extracted by mathematical models. The suffix “omics” is universally used in the clinic to define the concept of large data detection and extraction of valuable information and represents a revolution in traditional visual imaging technology. Its main application is the conversion of images into predictive models of phenotypic lesions, providing possible solutions to the limitation of current tools in pre- (at the diagnostic level, pathological and molecular classification), during- and post-treatment (prediction and management of response to treatment) procedures and determining prognosis [45]. Radiomics has mainly been developed on computed tomography (CT) images since this is the most universal diagnostic technique in this field. It is also being studied in magnetic resonance imaging and positron emission tomography and other imaging modalities with certain limitations.

The first step is to identify the characteristics of the regions of interest of the tumor. The main radiomics characteristics studied are:

- Structural:
  - Morphological, shape, and physical characteristics of the tumor.
  - Statistical. Gradients and textures. The latter predicts tumor heterogeneity and is most closely related to lung cancer outcomes.
  - Regionals. Clonal heterogeneity.
  - Model-based, fractal model that reflects the intrinsic shape of an object.
  - Wavelet features that identify the image in response to different spatial frequencies [46].

Radiogenomics focuses on defining the relationship between radiomic characteristics and genomic information, but more mature studies are still needed if this modality is to become useful in daily practice.

The first application of radiomics in lung cancer was published in 2014 by Aerts *et al*. [47], who showed that radiomics extracted information of prognostic value based on tumor gene expression.

Potential applications of radiomics in lung cancer:

- Diagnosis:
  - Evaluation of the lung nodule. Screening.
  - Pathological and molecular classification.
  - Patient management before surgery.
  - Prognosis:

- Survival analysis.
  - Detection of local recurrences.
  - Distant metastasis detection.

- Predicting responses to treatment
  - Choice and monitoring of targeted therapies.
  - Management of the response to RT and systemic treatments.
  - Monitoring of guided image-based radiation therapy.
  - Distinguishing between recurrence and lung damage from RT, a very important factor in post-SABR fibrosis.

In unresectable stage III NSCLC, treatment selection is complex due to a lack of predictive prior information. Radiomics is a useful tool for predicting response to treatments, monitoring targeted therapies and immunotherapy, monitoring image-guided RT, predicting recurrence, and detecting distant metastases.

Pre-treatment imaging is used to identify the association between quantitative characteristics with responses and outcomes after treatment is completed [45] (Table 4).

**Table 4:** Pre-treatment imaging radiomics in response assessment and treatment outcome prediction.

Studies images	Reference	Treatment	Patients (N)	Stage	Median follow-up	End points	Parameter related to results
PET only	Cook *et al.*	CRT	53	IB-III	21.2 m	RECIST	Coarseness
						PFS	Contrast
						Local PFS	Busyness
						OS	*P*<0.05
	Kang *et al.*	CRT	116	III	47.8 m	PFS	SUV max AUC-CSH
						LRFS	AUC-CSH
						DMFS	AUC-CSH
	Ohri *et al.*	CRT	201	IIB-III	22.6/20/6.2 m	OS	Textural feature: Sum Mean
	Carvahlo *et al.*	CRT	220	I-IIIB	1.47 years	OS	Relative volume above 80%
	Fried *et al.*	RT	195	III	37 m	OS risk stratification	Quantitative features with conventional PET metrics
CT only	Fried	CRT	91	III	59 m	OS	Combined texture features and conventional prognostic factors
						DM	
						LRC	
	Coroller *et al.*	CRT	182	II-III	23.7 m	DM	35 radiomic features
						OS	12 features
	Coroller *et al.*	CRT	127	II-III	41.8 m	Pathological response	GRD: 7 radiomics features
							pCR: 1 radiomic feature, rounder shape, heterogeneous texture
	Coroller *et al.*	CRT	85	II-III	40.2 m	Pathological response	pCR: 3 radiomics features, GRD: 2 radiomics features
	Aerts *et al.*	CRT/RT	647	I-IIIB	750 d	OS	238 features
	van Timmerman *et al.*	RT	288	I-IV	15 m/15 m/ 25.5 m	OS	13.3% radiomics features
	Song *et al.*	TKI	152	I-IV	9.5 m/10.2 m	PFS	2 texture features

AUC: Area under the receiver operating characteristic curve; AUC-CSH: Area under the curve of the cumulative; SUV-Volume histogram; CRT: Chemoradiotherapy; CT: Computed tomography; DFS: Disease-free survival; DM: Distant metastases; DMFS: Distant metastasis–free survival; DSS: Disease-specific survival; GRD: Gross residual disease; LR: Local recurrence; LRC: Local-regional control; LRFS: Locoregional recurrence-free survival; LRR: Loco-regional recurrence; OS: Overall survival; pCR: Pathologic complete remission; PET: Positron emission tomography; PFS: Progression-free survival; R2: Coefficient of determination; RECIST: Response Evaluation Criteria in Solid Tumors; RFS: Recurrence-free survival; rs: Spearman correlation; RT: Radiotherapy; SUV: Standardized uptake value; SUVmax: Maximum standardized uptake value; and TKI: Tyrosine kinase inhibitors

The study of changes in radiomics during and/or after treatments is called delta-radiomics. This application has been used to identify signs of recurrence or metastasis for the determination of prognosis and is an interesting development in image-guided RT, although so far, it has proven to be predictive only in colorectal cancer. The RECIST response has limitations in diversified clinical applications. Radiomic features, such as texture and volume changes, have the potential to better predict tumor responses and thus may be considered as new tumor response phenotypes that may provide diversified information in the future [48].

Today, we know that tumors develop from genetic mutations and that different models of interpatient behavior are expressed, explaining the different responses to treatments at the same stage of the disease. Furthermore, the value of biopsies, whether surgical or even complete resections, in pre-treatment diagnosis or during the course of the disease, is limited. Biopsies are often difficult to perform due to their location and they are incomplete studies of the tumor. Radiomics, however, non-invasively generates information on the entire lesion and can be conducted on repeated occasions during treatment and follow-up [49].

This methodology provides numerous significant advantages, but it also has its limitations, including the lack of standardization of equipment and processes and lack of consistency and robustness of data or integration with clinical factors such as age.

## 3. New RT technology in LA NSCLC

### 3.1. Introduction

Advances in molecular biology-targeted therapies and immunotherapy in NSCLC have led to an improvement in the prognosis of patients with advanced disease stages [50]. In parallel, significant progress has been made in the field of RT technology in NSCLC. Evidence suggests that technological innovation has also meant a breakthrough in the survival of these patients, although the data are derived from population registries [51-53] and retrospective studies [54]. Given the nature of the development and implementation of health technology, no data are available from controlled trials that have tested the potential advantages of these techniques against the older ones, nor are they likely to be developed [55].

Experience with 3-dimensional conformal RT (3DCRT) treatments revealed the relationship between irradiated lung volume and radio-induced toxicity [56,57]. Recently, dosimetric analysis of 3DCRT and intensity-modulated RT treatments in the RTOG 0617 trial [58] also demonstrated a correlation between doses received on the lungs and heart and survival of the patients. Therefore, a meticulous, accurate technique is essential to deliver a tumoricidal dose to the tumor while reducing the risk of adverse effects at OAR [59,60].

We review below the technological advances in the delivery of thoracic RT that allows an improved and more precise administration of RT, their clinical application and the benefit derived from the implementation of these techniques.

### 3.2. IGRT

IGRT consists of the use of imaging techniques to locate the position of the target volume at the time of radiation therapy, on a daily basis, to direct the radiation beam toward the actual location of the tumor by correcting the positioning of the patient [61].

Among the imaging techniques available, cone-beam CT (CBCT) with kV is the most attractive method for lung cancer RT [62] because it allows: (a) Sharper contrast to distinguish intrathoracic structures and better visualization of soft tissues, (b) the acquisition of images with 4-dimensional (4D) technology [63] with respiratory motion control, (c) monitoring of anatomical changes in the tumor and OAR, making it possible to check the initial RT plan and reassess if significant clinical differences are found, and (d) use of a non-invasive procedure for checking progress.

Daily CBCT image guidance for advanced NSCLC patients undergoing conventionally fractionated RT has shown effectiveness in reducing patient positioning inaccuracies, allowing a reduced PTV margin, which could potentially lead to a greater reduction in lung complications without compromising target coverage [64].

IGRT can be combined with tumor and breathing movement control as well as replanning techniques [65], as discussed below, for better optimization of radiation therapy delivery and improved clinical outcomes [66].

### 3.3. Breathing movement control

#### 3.3.1. 4DCT

This technique allows 3DCT reconstruction of the whole range of the tumor movement and its path correlated with the different phases of the respiratory cycle while maintaining image definition, thus minimizing the risk of failure in locating the target volume [67].

#### 3.3.2. Gating

Gating consists of activating the radiation beam on the accelerator when the tumor is placed in a position predefined by the operator during the simulation [68]; when the target leaves the region, the beam stops. The process is repeated as many times as necessary until the fraction is completed. Real-time tumor localization can be performed by indirect (optical recognition of the patient’s surface and respiratory movements [69]) or direct methods (inserting fiducial markers [70] or using 4DCT [71]). With this system, treatments can be significantly prolonged because several respiratory cycles are required to complete a session; furthermore, the patient’s collaboration is necessary (previous training is required), and a compromise must be reached between precise localization and amplitude of the trigger window (range between the “on” and “off” position), so as not to unduly lengthen the treatment time or jeopardize accuracy.

#### 3.3.3 Deep inspiration breath-hold (DIBH)

With this technique, the patient, with the help of a visual control system equipped with a spirometer, holds the inspiration (usually at 75% of its capacity) and keeps the target “static” in position for a limited time during RT [72]. DIBH differs from gating in that in the latter, the patient breathes normally and without pauses, whereas with DIBH, the patient is only treated when holding their breath. The advantage is that expanding the lung volume helps separate the intrathoracic structures, radiating less effective volume of lung and heart; however, the treatment time is prolonged and also requires active patient collaboration. The use of DIBH is poorly studied in lung cancer RT. Josipovic *et al*. [73] have published their prospective research on the extent of compliance and reproducibility of DIBH in patients with advanced lung cancer. Overall 72% (50/69) of the patients were compliant, and the target position was highly reproducible (deviations ≤3 mm in >90%).

#### 3.3.4. Tumor tracking

In this technique, the tumor is kept within the radiation beam throughout the respiratory cycle [74], without interruption, using surface, infrared, or invasive recognition systems [75] (fiducial markers or radiofrequency transponders). This RT delivery system requires fully robotic accelerators, so availability is very limited, and it is currently used only for SBRT [76].

As the estimation of the tumor position for RT planning will be based on the generation of a volume (ITV) [77] that includes the full length of the tumor path during the respiratory cycle, it could be combined with external systems that reduce the amplitude of diaphragmatic movement and therefore that of the tumor, making it more regular [78]. Moreover, reducing the fluctuation of the tumor position delays reconstruction artefacts in the CBCT. Abdominal compression is indicated when the target movement is >1 cm.

### 3.4. Adaptive RT (ART)

**Figure 1:**
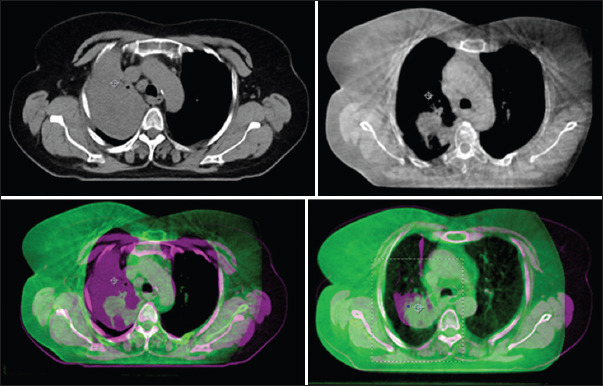
A 55-year-old woman diagnosed with non-small-cell lung cancer cT3 N2 M0 was treated with concurrent chemotherapy and radiation. This patient underwent intensity-modulated radiotherapy (RT) with image-guided RT (daily cone-beam computed tomography [CBCT]). Initial atelectasis improved until total resolution after 20 Gy. Adaptive RT (ART) was implemented by the treating physician to spare as much lung tissue as possible while keeping the tumor covered. The lower panel shows reference CBCT pre-ART (red) and post-ART (green).

As treatment progresses, changes occur that affect tumor volume, its position, or lung anatomy (e.g., atelectasis or pleural effusion), which may require replanning to avoid underdosing the PTV, or overexposure of healthy tissues, or both. Two studies have investigated the frequency of anatomical or tumor changes during lung RT using IGRT with daily CBCT. Kwit *et al*. [79] observed an overall incidence of changes of 72% (72% due to tumor, 28% anatomical). Appel *et al*. [80] reported an incidence of 74% due to changes in the tumor and 35.4% anatomical (some patients showed both events). An example is depicted in Figure 1.

The frequency of alterations that significantly affect clinical dosimetry and therefore warrant a reassessment of the RT plan has been estimated at around 9% [79] - 20% [80]. Kwint *et al*. [79] provided objective criteria to systematize the need to adjust the treatment. Experience indicates that with ART, it is possible to improve pulmonary and cardiac protection [80] while maintaining the therapeutic dose on the PTV. There is concern that replanning to a different volume of PTV smaller than the initial one will lead to increased recurrences because of the persistence of initial microscopic infiltration not covered by the reduced PTV [81]. In a prospective study [82] of the effect of ART on local control and toxicity, marginal relapse and out-of-field local failure were observed in 6% and 4% of patients, respectively; the main cause of local failure was recurrence in the PTV (20%). Overall, the incidence of local failure was not different from that reported with conventional techniques (31-38% in the RTOG 0617 trial [13]). Given that ART duplicates work, consumes resources, and may force treatment interruption, it is important to have triage tools to focus on those patients who really benefit [81,83] from replanning, and a dynamic and efficient work process to manage the care overload associated with ART [84].

### Conflicts of Interest

All the authors have no conflicts of interest.

### Financial Support

This research did not receive any specific grant from funding agencies in the public, commercial, or not-for-profit sectors.
